# Cancer control in Ghana: A narrative review in global context

**DOI:** 10.1016/j.heliyon.2020.e04564

**Published:** 2020-08-04

**Authors:** Kofi Boamah Mensah, Adwoa Bemah Boamah Mensah

**Affiliations:** a Department of Pharmacy Practice, Faculty of Pharmacy & Pharmaceutical Science, College of Health Science, Kwame Nkrumah University of Science & Technology, Ghana; b University of KwaZulu-Natal, Discipline of Pharmaceutical Sciences, College of Health Sciences, Westville Campus, University Road, Durban, South Africa; c Department of Nursing, College of Health Science, Kwame Nkrumah University of Science & Technology, Ghana

**Keywords:** Cancer research, Health sciences, Public health, Oncology, Clinical research, Cancer detection, Cancer control, Community pharmacists, Cancer prevention, Cancer

## Abstract

Globally, cancer is likely to be ranked as the leading cause of death among non-communicable diseases in the 21st century. In Ghana, estimates suggest that the disease is expected to increase continuously. The best way to address the increasing burden is through a comprehensive cancer control program. This paper presents an appraisal of the literature, reports and, studies that seek to highlight strategies for cancer control globally and in Ghana. In consideration of literature, a search of relevant databases (PubMed, Google Scholar, Cochrane Database of Systematic Reviews, Google, International organizations web pages, International reports, Ministry of Health of Ghana reports, and textbooks) was performed. A narrative review of the background information on this subject is provided to inform future research on cancer control. This review was conducted as part of a study to involve community pharmacists in cancer detection and prevention in Ghana.

## 1 Introduction

The majority of global deaths are now due to non-communicable diseases (NCDs) of which cancer is likely to be ranked as the leading cause of death and also the most likely barrier to increasing life expectance in every country of the world in the 21st century (Bray et al., 2018). Reports by World Health Organization (WHO) in 2015, indicate that cancer is the first or second major cause of death before age 70 years in 91 of 172 member countries, and also lead as third or fourth in an additional 22 member countries (World Health Organization, 2017).

Cancer prevalence and mortality are quickly increasing globally. Attributable reasons are complicated and may be due to ageing, growing population, late-stage presentation, inaccessible diagnosis, treatment, etc (IARC, 2018). Majority of cancers can currently be prevented by implementing evidence-based prevention strategies (World Health Organisation, 2018b) and early detection programs (IARC, 2018). Most cancers, such as lymphoma, thyroid, and testicular cancer have a high survival rate if diagnosed early and adequately treated (Kandola et al., 2018).

Prevention is known to provide a long-term cost-efficient strategy for cancer control (Stewart and Wild, 2014). Between, 30–50% of all cancer cases are preventable through avoidance of known risk factors (Vineis and Wild, 2014). The mortality rate of a proportion of the remaining 50% can be avoided by early detection (Ilbawi and Anderson, 2015). These detection and prevention programs form part of the basic structure of a cancer control program. Globally, the best way to address the burden of cancer is through a comprehensive cancer control program. According to the World Health Organization, this is a public health programme intended to decrease the number of cancer cases, mortality and improve the quality of life of cancer patients (World Health Organisation, 2015b). This can be achieved by implementing systematic, equitable and evidence-based prevention programs, timely detection, diagnosis, therapeutic and palliation using existing resources. Cancer control program can be instituted if it is well-conceived, well-managed, by all the relevant stakeholders, and at all levels of the decision-making process, in respective of limited available resources of the country.

Nevertheless, non-communicable diseases (NCDs) receive less than 3% of total donor development assistance for health ($503 million out of $22 billion per year) (Feigl, 2010), hence prioritization of cancer detection and prevention programs to reduce the burden of the disease in the global health agenda has been difficult. Evidence from developed countries shows that early detection and prevention programs are cost-efficient in reducing cancer death (Beaglehole et al., 2011). However, the establishment of these programs or strategies in developing countries is difficult due to competing health priorities, inadequate funding, limited human resources and lack of cancer awareness (Kingham et al., 2013).

In Ghana, there is a high burden of non-communicable disease, and among them, cancers have been projected to increase (Ministry of Health of Ghana, 2011). Though there is an establishment of a national cancer control plan, there is still an increasing incidence of cancers in the country due to lack of preparedness to cancer control (Ministry of Health of Ghana, 2011). In order to achieve NCDs Global Action Plan targets by 2025 and the Sustainable Development Goals (SDGs) by 2030, there must be evaluation of the current plan and greater emphasis on setting realistic cancer control programmes.

### 1.1 Aim

The purpose is to review literature and reports that seeks to highlight strategies for cancer control globally and in Ghana. This review was conducted as part of a study to involve community pharmacists in cancer detection and prevention in Ghana.

### 1.2 Design

A narrative review was undertaken to allow critical analysis of the literature published in books, published reports, newsletters, media reports and electronic or paper-based journal articles. This type of review is effective where the aggregation of data is difficult because different studies or fields are being analysed (Baumeister and Leary, 1997; Slavin, 1995). A narrative synthesis based on research studies on the role of community pharmacists in cancer control was included.

### 1.3 Search methods

In my consideration of literature, reports and studies on cancer control, I performed a search of relevant databases (Cochrane Database of Systematic Reviews, Google Scholar, Google, PubMed, International organizations web pages, International reports, media reports, Ministry of Health of Ghana reports and textbooks). The search words were cancer, cancer incidence, cancer screening, cancer control, cancer prevention, early detection, and community pharmacists. Information gathered from the generated references were used to write the narrative review if they were judged as providing relevant information.

## 2 Overview

### 2.1 Cancer

Cancer is a group of diseases in which a group of abnormal cells divide uncontrollably by ignoring the normal rules of cell division. The foundation of modern oncology rest on the principle that damage of the genetic apparatus of the cell is considered to be the primary cause of cancer. All mammalian cells share a similar molecular network that controls cell proliferation, differentiation and cell death. The prevailing theory underlining the genesis of cancer as evidenced by deep fundamental research is that, normal cells are transformed into cancers as a result of changes in this network held exclusively at the molecular, cellular, biochemical and genetic level (Kushlinskiĭ; Nemtsova, 2014; Weinberg, 2006).

### 2.2 Early signs and symptoms of cancer

A list of seven warning signs of cancer has been made publicly by the European Code Against Cancer (World Health Organization and International Agency for Research on Cancer, 2018). These signs are thickening or lump, unexplained weight loss, change in a wart or mole, change in bladder or bowel habits, unusual bleeding or discharge, nagging cough or hoarseness, and a sore that does not heal. Other agencies such as Imperial Cancer Research Fund (ICRF) and the Cancer Research Campaign (CRC) in the United Kingdom have also come out with a similar list of these signs and promoting it in the public arena. In the United States, the National Cancer Institute (NCI) and the American Cancer Society (ACS) have developed a similar list of warning signs. Except for the sign ‘unexplained weight loss,’ which is represented in the United States list of warning signs as ‘persistent indigestion or difficulty in swallowing’.

### 2.3 Risk factors of cancer

Research indicates that exposure to specific risk factors may increase the likelihood of developing cancer. However, most risk factors do not directly cause cancer or have a lower risk of causing cancer. Well-known risk factors for cancer are age, diet, alcohol, cancer-causing substances, obesity, chronic inflammation, hormones, immunosuppression, tobacco, infectious agents, radiation, sunlight (National Cancer Institute, 2015).

### 2.4 Cancer screening

According to the WHO, screening is the presumptive identification of unrecognized disease or defects by means of tests, examinations, or other procedures that can be applied rapidly (World Health Organisation, 2019). It is considered as a public health policy because it is applied to the population. One of the critical objectives of cancer screening is to reduce mortality and promote the quality of life by detecting cancers early and by adequately treating them (Sankaranarayanan, 2014). Several screening tests and recommendations have been known to detect cancer early and reduce mortality. In the 1980s, the America Cancer Society (ACS) introduced and have regularly updated their cancer screening guidelines. Other organizations have also developed similar guidelines, but sometimes differed slightly or entirely to particular available technology, ages to start and stop screening, screening intervals and available data (Smith et al., 2012). Some of the screening modalities that have been shown to reduce cancer mortality by the ACS are mammography, Colonoscopy, sigmoidoscopy, high-sensitivity fecal occult blood tests (FOBTs), Papanicolaou and human papillomavirus (HPV) test, Low-dose helical computed tomography, Papanicolaou test. Others also include alpha-fetoprotein blood test, breast MRI, clinical breast exams and regular breast self-exams, prostate-specific antigen (PSA) test, skin examination, transvaginal ultrasound and virtual colonoscopy and CA-125 test.

### 2.5 Global cancer control strategy

In 2005, member countries of the WHO at its Fifty-eighth assembly (58th World Health Assembly) accepted a resolution to advocate for the fight against cancer by establishing and promoting programs with objectives of reducing the prevalence and mortality of the disease while improving the wellbeing of the patient and their caregivers. According to the resolution, this is achievable through the use of empirical research based on prevention, early detection, diagnosis, treatment, rehabilitation and palliative care (World Health Organization, 2005).

In 2008, WHO in response to WHA 58 resolution, developed guidelines for cancer control in six different components namely planning, prevention, early detection, diagnosis and Treatment, palliative care and Policy and Advocacy (World Health Organization, 2008). Within this same year, the regional office of WHO for Africa came out with a publication on programs for cancer prevention and control in Africa. The program highlighted the need for a comprehensive and guidance framework on cancer control that can be incorporated into the national health programs of member countries. Areas of priority included were the development of policies, adequate resources, training of health staffs, provision of proper infrastructure, surveillance and research, etc. (WHO Regional Office for Africa, 04/06/2008).

In 2015, the WHO NCD Country Capacity Survey (CCS) was done to collect comprehensive information on how member countries are faring in dealing with NCDs and with greater attention on cancer control (World Health Organisation, 2015a). A total of 177 out of 194-member countries took part in the survey. Results of the study indicated differences and considerable gaps in the present state of complete cancer control. Even though most member countries have developed policies on cancer control, more work was expected to make sure that these policies become functional.

One of the priorities identified in this survey was for member countries to improve their national health information structures and national cancer registry. These were important for policy-making, patterns in cancer control, quality and health access. Another vital area recognized in the survey was building capacity at the primary care level (e.g. community pharmacies, clinics, maternity homes, herbalists, general practitioner's office) to be able to identify early symptoms of cancer and also improve prompt referral of patients. Remarkable deficiencies in cancer diagnosis and care in the public sector was identified especially in lower-income countries where lower than 30% of member countries have accessibility to cancer management centers or services. To achieve the aim of reducing cancer mortality, these deficiencies were to be addressed with empirical evidence and cost-efficient strategies that help early recognition and access to care and palliative care for everyone (World Health Organisation, 2015a).

In 2017, another WHO NCD CCS was done to measure developments in member countries capacity building for cancer control (World Health Organisation, 2018a). The 2017 report indicated that breast and cervical cancer screening programmes were generally accessible in almost three-quarters of countries, but colon cancer screening programmes were far less widely available (39% of countries). Most screening programmes in lower- and middle-income countries were not reaching a substantial portion of the target population but rather reaching 50% or less of the target population. Most (61–67%) of countries reported integrating early detection programmes or guidelines for breast and cervical cancer into primary healthcare services. Just 37% of member countries had a similar program for colon cancer. Also, 60–65% of countries had a clearly defined referral system used for breast and cervical cancer, and only 44% had such a system for colon cancer.

In conclusion of the report, cancer screening programmes are still failing in reaching most of their target populations, and many remain opportunistic. Significant work is needed to improve the program in low-income countries. Overall findings of the 2017 NCD CCS indicate that, though considerable progress has been made in nations responses to prevent and address cancer, a wide range of opportunities for improvement still exist (World Health Organisation, 2018a). Assessment of 2017 WHO NCD CCS progress will be done in 2019.

### 2.6 Introduction to cancer control in Ghana

Ghana lacks credible data on cancers due to insufficient cancer registries. Accessible data on cancers are from health organizations records. Though organizational records are scanty in terms of national coverage, the extent of disease, statistical limitations, these records indicate that cancers are now becoming the primary cause of morbidity and mortality in the country (Laryea et al., 2014). As part of the requirements to achieve the Millennium Development Goals (MDGs), the health ministry established the Health Sector Medium Term Development Plan (HSMTDP) to intensifying measures to prevent and control communicable and non-communicable diseases (Ministry of Health of Ghana, 2011).

In a similar event, the 58th World Health Assembly (WHA) pass a resolution for member countries to intensify efforts in reducing the burden of cancer by establishing and strengthening cancer control programs (Kanavos, 2006). In order to accomplish this objective in the country, a National Cancer Control Steering Committee (NCCSC) was set up to recommend and direct stakeholders in the establishment and implementation of a nationwide cancer control program with a focus on WHO, 2008, published guidelines for effective cancer control (Ministry of Health of Ghana, 2011).

Major stakeholders such as Ghana Health Service (GHS) with their partners, both local and international initiated cancer advocacy campaigns with a focus on simple lifestyle modifications such as smoke cessation, exercising and having regular medical checks to reduce cancer occurrence. Considering the strategies outlined in the WHA 58 resolution, Ghana needs a comprehensive approach.

### 2.7 Cancer detection in Ghana

Evidence from randomized trials has shown that cure from early stage cancer is high (Levin et al., 2003). The essential tool for early cancer detection is the availability of effective, cheap, simple and safe screening examinations. The most researched screening tests include mammography (for breast cancer screening), Pap cytology test (for cervical cancer screening) and fecal occult blood test (for colorectal cancer screening).

In Ghana, early diagnosis and screening are part of the strategies employed in early cancer detection. For breast cancer, self-breast examination and clinical breast examination are the significant practices mostly employed (Naku Ghartey Jnr et al., 2016).

Human Papilloma Virus infection (HPV), which is acquired through sexual intercourse, is known to be the primary cause of cervical cancer (Bekkers et al., 2006). Early detection of the disease in many hospitals in the capital city, Accra, is achieved by examination of the cervix with acetic acid (VIA) or by Papanicolau (Pap) smear (Adadevoh and Forkouh, 1993; Sanghvi et al., 2008). Studies have shown that black people are at high risk of developing cancer of the prostate than Caucasian (Polednak, 2003). Epidemiological studies have revealed the prevalence of prostate cancer in Ghana as >200/100,000 compared to other West African countries such as Nigeria (127/100,000) and Cameroun (130/100,000) (Angwafo et al., 2003). There is evidence suggesting that population-based screening may decrease prostate cancer death (Van Leeuwen et al., 2013). However, there are uncertainties about its reliability (Gottlieb, 2003). The European Randomised Study of Screening for Prostate Cancer (ERSPC) reported a reduction of only 9% in the long term risk of prostate cancer-related mortality (Schröder et al., 2014). Prostate, Lung, Colorectal, and Ovarian Cancer Screening Trial, also reported no significant decrease in prostate cancer-related mortality (Andriole et al., 2009). Again, other systematic reviews have also reported that PSA screening has limited or no effect on the overall disease and prostate cancer-related mortality (Ilic et al., 2013).

There is low awareness among Ghanaian men on prostate cancer, its signs and symptoms (Yeboah-Asiamah and Bernard, 2015; Otoo, 2010). However, precautionary measure for detection of the disease through early non-invasive screening using prostate-specific-antigen (PSA) is practiced.

### 2.8 Cancer risk in Ghana

The leading causes of certain cancers have been known to be as a result of exposure to certain risk factors such as smoking (Danaei et al., 2005). Removal or reduction in exposure to these risk factors can help prevent and reduce the burden of the cancers.

This intervention is the easiest and cost-effective way of improving public health. These risk factors can be grouped as behavioral, lifestyle and environmental factors. Effective prevention programs should focus on modifiable risk factors with a significant impact on cancer burden.

Findings from a study by Jemal et al. indicates that, strategies and policies aimed at certain modifiable risk factors such as tobacco prevention and weight managing other than any other intervention in developing countries could be used to reduce the burden of cancer (Jemal et al., 2012). The incidence of many of the behavioural risk factors in Ghana is increasing. A study in Ghana reported 62.3% of women are obese and overweight. This same study reported that more than 85% of Ghanaians consume less than the recommended five daily servings of fruits and vegetables (Hill et al., 2007). A risk factor surveillance which was done by the Ghana health service in April 2010 in the capital city, Accra, reported 60.2% prevalence in obesity and overweight among men and women. Also, 27.4% of school children between the ages of 13–15 years in Ghana are physically inactive (Owusu, 2007).

It is essential to know that, worldwide, about 10–16% of colon, rectal and breast cancer cases can be attributed to physical inactivity (World Health Organization, 2002). Smoking is one of the most critical risk factors for cancer under lifestyle modification. Smoking in men declined from 10.7% in 2003 to 8.1% in 2008 in Ghana (Noguchi Memorial Institute for Medical Research, 2004). Research shows that 2.6% of the total annual household expenditure of a Ghanaian is spent on alcoholic drinks and 1.6% on tobacco in the year 2005 (Ministry of Health of Ghana, 2011). Further research shows that Ghanaians living in rural areas account for a little over 65% of the yearly expenditure on alcoholic drinks and tobacco in Ghana (Ministry of Health of Ghana, 2011). Also, a study in Ghana indicates that the harmful effects of alcohol consumption range from 3% to 6% (Amoah, 2003).

Over 150 chemical or biological agents encountered at the workplace have been known to be carcinogenic (Driscoll et al., 2004). Worldwide, 10.3% of lung, trachea and bronchus cancer cases occurred as a result of occupational exposures (Driscoll et al., 2004). In 2017, 40.65% of the population were in the agricultural sector (The Statistics Portal, 2017); hence, there may be a high probability of exposure to agrochemicals and other chemical or biological agents. Again, small scale mining is widespread in some geographical regions of Ghana (Owusu-Nimo et al., 2018). Majority of the citizens engaged in this practice, do it illegally. There is a high probability of exposure of these miners and the environment to chemical or biological agents which may be carcinogenic.

### 2.9 Strategies for cancer control in Ghana

The Ministry of Health of Ghana during its celebration of world cancer day in 2015 inaugurated the national strategy for cancer control. The strategy was prepared with expert support from WHO and other international partners to reduce cancer incidence in Ghana (WHO Regional Office for Africa). The strategies to be employed to accomplish this objective are, promotion of healthy diet, physical activities, screening and early detection, palliative care, treatment, cancer registry and research, partnerships, and legislation on cancer risk substances (e.g., Tobacco).

The National Strategy for Cancer Control in Ghana (2012–2016) proposed that ‘breast cancer screening should be taught in school as a means of creating breast cancer awareness to help in early detection of the disease’ (Kudzawu et al., 2016). This program was proposed to start with school pupil 16 years of age in every school in the country. This program has not yet been executed (Ministry of Health of Ghana, 2011). Another strategic plan of the Ministry of Health of Ghana was also to train health professionals to start clinical breast examination in all health centers in the country. Facilities such as Komfo Anokye Teaching Hospital has a breast care center with trained health personnel's that offer clinical breast examination service to the public.

As part of government measures in the fight against gynaecological cancers, a policy on it was established as part of the National Reproductive Health Policy of the country. The policy advised screening for cancer of the cervix by Visual Inspection with Acetic Acid (VIA), Papanicolaou test for women aged 45 and above and cryotherapy of women (25–45 years) with pre-cancerous lesions (Ministry of Health of Ghana, 2011). Again, gynaecological cancers screening has also been integrated into the existing health programs such as the reproductive health programs (Family planning and sexually transmitted infections management services). Primary healthcare givers who offer this service, also provide cervical cancer screening and vaccination services for individuals who seek family planning, sexually transmitted diseases management services.

An international non-profit health organization associated with Johns Hopkins University which was at first called the Johns Hopkins Program for International Education in Gynecology and Obstetrics, but is now referred to only as JHPIEGO, with one of its focus being on reproductive health, started a national gynecological cancer screening program in Ghana called the JHPIEGO Safe PROJECT. The program began in March 2000 and 2002, with the main focus on cervical cancer prevention. It was later referred to as a JHPIEGO Cervical Cancer Prevention (CECAP) project (Odoi-Agyarko, 2003). The program initially implemented visual inspection screening pilot programs at two centers in Accra, one urban health facility, and one rural health center, and later, other centers were established. Preliminary findings from the project, after more than a year from the first two centers, show that pre-cancerous lesions detected in the urban and rural health facilities were 11.1% and 6.6%, respectively (Odoi-Agyarko, 2003). This early detection of pre-cancerous lesions indicates early treatment and prevention of cervical cancer, which would have increased the burden of the disease in the country. The government of Ghana intended to adopt this as a national screening model for cancer control (Abotchie and Shokar, 2009). The strategy for adaptation of CECAP was to divide the country into three zones. These three zones will become the focal points for expanding down to the sub-district levels. As part of the program, master trainers will be pointed and trained for each zone. These master trainers will train clinical trainers who will train service providers. This was to make available service provision at the sub-district level within five years of implementation of the program (Abotchie and Shokar, 2009). Currently, only a few centers which were part of the project are still active post project.

The Ministry of Health of Ghana according to its strategy for gastrointestinal cancer control is promoting public knowledge on early warning signs of gastrointestinal cancer and the need for cancer screening (Ministry of Health of Ghana, 2011). According to the strategy, health personnel at all stages of the healthcare structure would undergo training on gastrointestinal cancer screening techniques. The author believes training of all health workers will not be a sustainable strategy because not all of them are directly involved with patients. Primary health workers who are the first point of call to the public for their healthcare needs are the very essential personnel's who can sustain this strategy.

The strategic plan in place to control adult blood cancers in Ghana is through the creation of public awareness on the signs and symptoms of the various types of blood cancers since there is no population-based screening test for early detection of the disease.

To the best of the author's knowledge, there is no existence of a national childhood cancer control program in Ghana. Non-profit organizations and individual advocates have recommended public health education as a strategy to reduce the burden of childhood cancers. A non-profit organization, Lifeline for Childhood Cancer, Ghana, was formed to create awareness about childhood cancer among health workers and the general public (GhanaWeb, 2018). Also, the Programme Administrator of a Non-Communicable Disease Control Programme (NCD) at the Korle-Bu Teaching hospital in Ghana also recommended that ‘related authorities should create awareness through educational programmes on the rising cases of cancer among children, and for the public, particularly mothers, to take precautionary measures to avoid the occurrence of cancers among their children’ (GhanaWeb, 2017).

Ghana became the 39th nation to endorse the World Health Organization's Framework Convention on Tobacco Control (FCTC) which presented an opportunity for legislation on tobacco use (Owusu-Dabo et al., 2010; Wellington et al., 2009). The Parliament of Ghana then approved an upward adjustment in the price of tobacco and other related products as part of efforts to decrease tobacco use and also pass the tobacco bill into law (Ankrah, 2019). This bill prohibits sales of tobacco to minors (Owusu-Dabo et al., 2010) and alcohol to children under 18 years (Mensah-Kufuor, 2018). The passage of this bill into law is one of the strategies used by the Ministry of Health to decrease the risk of certain cancers such as lung, breast, cervical and prostate cancer.

At least six viruses are known to cause cancer, namely Epstein-Barr virus (EBV), Hepatitis B virus (HBV), Hepatitis C virus (HCV), several Human Papillomavirus (HPV) types, Human T-cell Lymphotropic Virus type I (HTLV-I) and Human Immunodeficiency Virus type I (HIV–I) (Boccardo and Villa, 2007). Prevention strategies are complicated in cancers of an infective cause because infected cells may maintain the state of latency for years before turning on the oncogenic pathway (Butel, 2000). However, knowledge about the spread of these infections is known. Hence, this knowledge provides directions for prevention. Some of these infections are transmitted sexually, thus practising safe sex, abstinence, are the approach towards infection control (Alexander et al., 2012). Another intervention lies in the development of antiviral vaccines. (HPV) and HBV vaccines are registered and available for use in Ghana. Also, since the year 2000, children receive HBV vaccine as part of the country childhood immunization program. Also, as part of the country strategy for cancer control, awareness programs have been instituted or in place to educate the public about cervical cancer and hepatitis B infection and the need for vaccination. Hepatitis C has no vaccine; hence, there is a need for an intense awareness program on it. For this program to be effective nationwide, primary healthcare givers who are the front-liners to offer health information should be knowledgeable to educate the public.

### 2.10 The role of community pharmacist in cancer control

The practice of pharmacy has shifted from the traditional dispensing activities to patient focus activities or patient-oriented services (El Hajj and Hamid, 2011; Wiedenmayer et al., 2006). This emerging role of community pharmacists has pushed them to include cancer control activities into their regular practice.

A study done in United States indicated that pharmacists are involved in skin cancer prevention by educating patients and also the public on the disease (Mayer et al., 1998). They do this by counselling patients on related risk factors and even choice of sunscreen creams to be used. An interventional study by Potter et al. indicated that patients prefer and are responsive to cancer screening programs such as colorectal cancer screening organized by community pharmacists (Potter et al., 2010). This study shows public opinion on cancer screening activities or services organize by community pharmacists. Another study assessing women preference on their choice of setting to undergo mammography indicated that majority preferred the community pharmacy setup due to its convenience and accessibility (Gupta et al., 2012). A systematic review by Lindsey et al., on the role of community pharmacist in early cancer detection concluded that, six (6) studies out of the twelve (12) relevant studies identified indicated that community pharmacists have an effect on patients’ behaviour on cancer screening activities (Lindsey et al., 2015). The review concluded that screening programs in community pharmacies are possible.

In Ghana, most cancer patients seek care on their own (Ohene-Yeboah and Adjei, 2012; Thomas et al., 2017) and from the view of the authors, the health-seeking behaviour practice of Ghanaians are, most of them seek-healthcare first from a community pharmacy. Hence, from the main author's clinical practice as a specialist oncology pharmacist in Ghana, he observed that some of the cancer cases that are presented late had frequently spent time with the community pharmacist at the community pharmacy where signs and symptoms of cancer are managed as common or general symptoms. This causes a delay and lead to advanced stage disease. A recent study done in Ghana among community pharmacist concluded that community pharmacists would play an essential role in cancer health promotion through their professional practice (Mensah et al., 2018), though a pilot study reported inadequate knowledge among community pharmacists in Ghana (Mensah et al., 2019). Knowledge gap mostly leads to late referral to a secondary or specialist healthcare and also a late presentation of cancer. Hence if the community pharmacist knowledge gap is addressed, they may be the best primary healthcare provider to start cancer control practices in Ghana.

## 3 Conclusion

The strategy or programs for cancer control in Ghana has to be strengthened. The suggested approach of involving all healthcare personnel's in cancer screening will have to be based on preliminary investigations. Nonetheless, studies, reports, communications, employed in this review indicates that the essential pillars of cancer control are early cancer detection and prevention program. The vital aspect of this program involves educating the public on cancer risk factors, screening tests for cancer, signs and symptoms of cancer. With the increasing burden of the disease, secondary healthcare providers will have to depend on primary healthcare providers to perform this important role. Primary healthcare workers are the first contact for the public in the provision of the healthcare needs of the general public. Community pharmacists are known to be the most accessible primary healthcare providers who provide a free consultation, located in the communities, have extended working hours, easily accessible. Also, most patients preferred the community pharmacy setting and accept cancer control programs performed at the community pharmacy. Hence these healthcare workers may be the most suitable professionals to start early cancer detection and prevention program. It is thus vital for these healthcare providers to be aware of cancer to be able to perform this role effectively. It is then essential to conduct a large-scale study to access the awareness of community pharmacists on cancer, risk factors, signs and symptoms globally and in Ghana.

## Declarations

### Author contribution statement

All authors listed have significantly contributed to the development and the writing of this article.

### Funding statement

This research did not receive any specific grant from funding agencies in the public, commercial, or not-for-profit sectors.

### Competing interest statement

The authors declare no conflict of interest.

### Additional information

No additional information is available for this paper.
